# Displacement of Native FXYD Protein From Na^+^/K^+^-ATPase With Novel FXYD Peptide Derivatives: Effects on Doxorubicin Cytotoxicity

**DOI:** 10.3389/fonc.2022.859216

**Published:** 2022-03-17

**Authors:** Chia-Chi Liu, Yeon Jae Kim, Rachel Teh, Alvaro Garcia, Elisha J. Hamilton, Flemming Cornelius, Robert C. Baxter, Helge H. Rasmussen

**Affiliations:** ^1^ North Shore Heart Research Group, Kolling Medical Research Institute, University of Sydney, St Leonards, NSW, Australia; ^2^ School of Chemistry, University of Sydney, Camperdown, NSW, Australia; ^3^ Department of Biomedicine, University of Aarhus, Aarhus C, Denmark; ^4^ Hormones and Cancer Laboratories, Kolling Institute, University of Sydney, St Leonards, NSW, Australia; ^5^ Department of Cardiology, Royal North Shore Hospital, St Leonards, NSW, Australia

**Keywords:** breast cancer, pancreatic cancer, peptide therapy, membrane transport, oxidative stress, dysadherin

## Abstract

The seven mammalian FXYD proteins associate closely with α/β heterodimers of Na^+^/K^+^-ATPase. Most of them protect the β1 subunit against glutathionylation, an oxidative modification that destabilizes the heterodimer and inhibits Na^+^/K^+^-ATPase activity. A specific cysteine (Cys) residue of FXYD proteins is critical for such protection. One of the FXYD proteins, FXYD3, confers treatment resistance when overexpressed in cancer cells. We developed two FXYD3 peptide derivatives. FXYD3-pep CKCK retained the Cys residue that can undergo glutathionylation and that is critical for protecting the β1 subunit against glutathionylation. FXYD3-pep SKSK had all Cys residues mutated to Serine (Ser). The chemotherapeutic doxorubicin induces oxidative stress, and suppression of FXYD3 with siRNA in pancreatic- and breast cancer cells that strongly express FXYD3 increased doxorubicin-induced cytotoxicity. Exposing cells to FXYD3-pep SKSK decreased co-immunoprecipitation of FXYD3 with the α1 Na^+^/K^+^-ATPase subunit. FXYD3-pep SKSK reproduced the increase in doxorubicin-induced cytotoxicity seen after FXYD3 siRNA transfection in pancreatic- and breast cancer cells that overexpressed FXYD3, while FXYD3-pep CKCK boosted the native protein’s protection against doxorubicin. Neither peptide affected doxorubicin’s cytotoxicity on cells with no or low FXYD3 expression. Fluorescently labeled FXYD3-pep SKSK was detected in a perinuclear distribution in the cells overexpressing FXYD3, and plasmalemmal Na^+^/K^+^-ATPase turnover could not be implicated in the increased sensitivity to doxorubicin that FXYD3-pep SKSK caused. FXYD peptide derivatives allow rapid elimination or amplification of native FXYD protein function. Here, their effects implicate the Cys residue that is critical for countering β1 subunit glutathionylation in the augmentation of cytotoxicity with siRNA-induced downregulation of FXYD3.

## Introduction

The 7-member mammalian FXYD protein family is expressed in a tissue-specific manner (1). Its members associate closely with the Na^+^/K^+^-ATPase, and most of them protect the Na^+^/K^+^-ATPase β1 subunit against glutathionylation, an oxidative modification in which the cytosolic tri-peptide glutathione (GSH) binds to a free SH group of a cysteine (Cys) residue in a protein. Glutathionylation of the β1 subunit compromises the structural integrity of the α1/β1 heterodimer of Na^+^/K^+^-ATPase and inhibits its function. Countering this, FXYD proteins can stabilize Na^+^/K^+^-ATPase structure and function (2).

A specific Cys residue bracketed by basic amino acids and conserved across most members of the FXYD protein family is critical for a FXYD protein’s protection against β1 subunit glutathionylation. The cellular expression of FXYD proteins that have the specific Cys residue is protective against oxidative stress-induced β1 subunit glutathionylation and Na^+^/K^+^-ATPase inhibition, while mutating the residue eliminates the protective effect of the expressed protein (2). The critical role of Cys residues is also evident from the effects of exposing cardiac myocytes to recombinant FXYD proteins with or without the Cys residue (2).

A brief (15 min) exposure of myocytes to recombinant FXYD3 protein displaces the native FXYD1 protein. Both the exogenous and the native protein have the Cys residue critical for the protective effect against β1 subunit glutathionylation, and the additional exogenous supply of FXYD3 counters β1 subunit glutathionylation induced by oxidative stress. In parallel, it counters sarcolemmal Na^+^/K^+^-ATPase inhibition. Exogenous recombinant FXYD1 has similar effects on Na^+^/K^+^-ATPase function. However, while a recombinant FXYD3 protein with Cys residues mutated to serine also displaces native FXYD1 in myocytes, it is not protective against β1 subunit glutathionylation or Na^+^/K^+^-ATPase inhibition (2).

Only the transmembrane domain and the extracellular PFXYD motif near the membrane leaflet have known bonds to α- and β Na^+^/K^+^-ATPase subunits (3, 4). Other than the PFXYD motif and the motif that includes the Cys residue critical for countering β1 subunit glutathionylation, extracellular and cytoplasmic domains are poorly conserved across the FXYD protein family (5). Since they are not expected to affect β1 subunit glutathionylation, we designed 36 amino acid peptide analogues of the Cys-preserved FXYD3 protein and its Cys-mutated (Cys →Ser) derivative without these domains. We refer to these peptides as FXYD3-pep CKCK and FXYD3-pep SKSK.

FXYD3 is often highly expressed in cancers, particularly in those of the pancreas (6), prostate (7), and breast (8). Suppression of FXYD3 with siRNA in cultured human breast cancer cells that overexpress FXYD3 augments cytotoxicity of doxorubicin (Dox) (9). Dox induces oxidative stress (10), and the augmentation of Dox cytotoxicity with downregulation of FXYD3 was associated with an increase in β1 Na^+^/K^+^-ATPase subunit glutathionylation (9). If augmentation of cytotoxicity with FXYD3 downregulation depends on the FXYD Cys residue that is critical for countering β1 subunit glutathionylation, Cys-mutated FXYD3-pep SKSK but not FXYD3-pep CKCK should reproduce the augmentation. We have examined the effects of FXYD3-pep SKSK or FXYD3-pep CKCK on Dox-induced cytotoxicity in breast and pancreatic cancer cells that do or do not overexpress FXYD3.

## Materials and Methods

### Cell Cultures

Human pancreatic cancer cells BxPC-3, Panc-1, non-transformed human mammary epithelial MCF-10A cells, and human breast cancer cells MCF-7 and MDA-MB-468 were obtained from the American Type Culture Collection (ATCC, Manassas, VA, USA). The cells were then cultured as previously described (6, 9, 11). All cells were used within 20 passages of thawing and were free of mycoplasma contamination as ascertained by mycoplasma PCR detection (12).

### FXYD3 siRNA Transfection

A human siRNA against FXYD3 (consisting of pools of three to five target-specific 19–25-nt siRNAs designed to knock down gene expression, sc-60665) and the non-silencing control (control siRNA-A, sc-37007) were purchased from Santa Cruz Biotechnology (Dallas, TX, USA). Cells were transfected using the siRNA Transfection Reagent/Medium (Santa Cruz Biotechnology) according to the manufacturer’s instructions. The FXYD3 mRNA expression level was quantified by real-time polymerase chain reaction (RT-PCR) as described previously (9). FXYD3 protein abundance was measured by Western blotting (9).

### Susceptibility to Glutathionylation of FXYD3 Peptide Derivatives

FXYD3 peptide derivatives were synthesized by Mimotopes Pty Ltd., Australia. Since an FXYD protein’s susceptibility to glutathionylation is essential for the protein to counter glutathionylation of the β1 subunit (2), we ascertained that FXYD3-pep CKCK but not FXYD3-pep SKSK is susceptible to glutathionylation. Glutathionylation was performed by disulfide exchange with oxidized glutathione (GSSG) (13). Peptides were incubated with 10 mM GSSG (30 min) or 10 mM GSH/100 mM hydrogen peroxide (H_2_O_2,_ 30 _min_). Dithiothreitol (DTT) was added for disruption of protein disulfide bonds. N-Ethylmaleimide (NEM, 5 mM for 5 min on ice) was also added to block free thiols and minimize exchange between free thiols and oxidized thiols.

### Fluorescent Confocal Microscopy

The extracellular N-terminal of customized FXYD3-pep SKSK was tagged with fluorescent tetramethylrhodamine (TRITC) to visualize the distribution of FXYD peptide derivatives in cells. BxPC-3 cells grown to 70% confluence were exposed to 1 µM FXYD3-pep SKSK with or without TRITC-tag. Cells were exposed to the peptide for 2 h, the media removed, peptide-free fresh media added, and cells incubated for a further 24 h. They were then fixed in 3.7% paraformaldehyde, washed, and mounted on non-coated color frost slides in Fluoroshield mounting medium with DAPI (ab104139, Abcam, Cambridge, MA, USA) and examined under a laser scanning confocal microscope (Leica TCS SP5, Wetzlar, Germany). The excitation wavelength was 543 nm, and the emission wavelength was 572 nm. The fluorescence images were obtained using constant settings of scanning speed, pinhole diameter, and voltage gain.

### Cell Viability and Apoptosis

Cell metabolic activity was assayed as described (14) by estimating the reduction of XTT (2,3-bis(2-methoxy-4-nitro-5-sulfophenyl)-2*H*-tetrazolium-5-carboxyanilide), using a commercially available kit (Cell Signaling Technology, Danvers, MA, USA) according to the manufacturer’s instructions. Metabolic activity was used as a surrogate for viability. For each set of experimental groups, we performed 5 experiments with 4 replicates. Caspase-3-like activity is increased through a protease cascade during the early stage of apoptosis (15), and we measured activities of Caspase 3/7 (DEVDase) using the caspase fluorogenic substrate (Calbiochem, San Diego, CA, USA) as described (9).

### Co-Immunoprecipitation

After solubilization in RIPA lysis buffer, protein extracts (0.5–1 mg) from cells were precleared and incubated overnight at 4°C with anti-α_1_ subunit Na^+^-K^+^ ATPase (D4Y7E; Cell Signaling Technology, USA), anti-FXYD3 (ab205534; Abcam, UK), or anti-β1 subunit (D6U8Q; Cell Signaling Technology, USA) Na^+^/K^+^-ATPase antibodies followed by precipitation for 2 h at 4°C with protein A/G plus agarose-coated beads (Abcam, UK). Sample buffer was added, the mixture was boiled for 5 min and sedimented, and the supernatant was used for immunoblotting.

### Western Blot

Western blot analysis was performed as previously described (9). The following primary antibodies were used: Na^+^-K^+^ ATPase α1 subunit (05-369; Merck Millipore, Burlington, MA, USA), Na^+^-K^+^-ATPase β1 subunit (A278; Merck Millipore, USA), FXYD3 (OTI1D1; Sigma-Aldrich, AUS), or GSH (recognizing GS-S-proteins purchased from Virogen, Watertown, MA, USA). For a loading control, GAPDH was detected using the anti-GAPDH monoclonal antibody (G8795; Sigma-Aldrich, Sydney, Australia). Each presented immunoblot is representative of separate experiments as indicated in text. The band densities were quantified by densitometry (Image Lab™, 6.0.1 Bio-Lab Laboratories, Inc., Hercules, CA, USA).

### Measurement of Plasmalemmal Na^+^/K^+^-ATPase Activity

The whole-cell patch-clamp technique was used to measure plasmalemmal electrogenic Na^+^/K^+^-ATPase pump current (I_p_, arising from the 3:2 ratio of intracellular Na^+^ pumped out of cells in exchange for extracellular K^+^ pumped in). Solutions and voltage-clamp protocols were designed to minimize non-pump currents. Patch pipette solutions that perfuse the intracellular compartment after the whole-cell voltage clamp configuration is established contained the following (in mM): 1 NaH_2_PO_4_, 5 HEPES, 5 EGTA, 2 MgATP, 86 Na^+^-glutamate, and 70 tetramethylammonium chloride. The solution was titrated to a pH of 7.2 at 22°C using 2 M NaOH. The final concentration of Na^+^ was 100 mM which causes near-maximal plasmalemmal Na^+^/K^+^-ATPase activation at intracellular sites.

Cells were initially superfused with modified Tyrode’s solution containing the following (in mM): 140 NaCl, 5.6 KCl, 2.16 CaCl_2_, 0.44 NaH_2_PO_4_, 10 glucose, 1.0 MgCl_2_, and 10 HEPES. The solution was titrated to a pH of 7.55 at 22°C with NaOH. After the whole-cell configuration was established, we switched the superfusate to one that was nominally Ca^2+^-free and contained 0.2 mM CdCl_2_ and 2 mM BaCl_2_. Cd^2+^ was included to block Ca^2+^ channel conductance and inhibit Na^+^-Ca^2+^ exchange (16), while Ba^2+^ was included to block K^+^ channels. Cells were voltage clamped at 0 mV to inactivate voltage-sensitive Na^+^ channels (17) and L-type Ca^2+^ channels (18, 19).

I_p_ was identified as the inward shift in holding current with exposure to K^+^-free extracellular solutions to eliminate Na^+^/K^+^-ATPase activation at extracellular sites, as described previously for measurements in cardiac myocytes (20). We switched to the K^+^-free superfusate 2–3 minutes after the whole-cell configuration had been established. Na^+^/K^+^-ATPase pump currents are small relative to other membrane currents, and it is important for their accurate measurement that holding currents are stable before and after the switch from K^+^-containing to K^+^-free solutions. As for measurement of I_p_ in cardiac myocytes (20), we used predetermined criteria for stability of holding currents before and after changing to K^+^-free extracellular solution. I_p_ was normalized for cell membrane capacitance and hence cell size.

### Statistical Analysis

Results are presented as mean ± standard deviation (SD). The IC50 values for Dox in cell viability studies were calculated by GraphPad Prism. Statistical comparisons were made with a Mann–Whitney test, ANOVA, and repeated-measure ANOVA with Geisser–Greenhouse epsilon correction. p < 0.05 was considered statistically significant.

## Results

### FXYD3-siRNA Transfection Augments Dox-Induced Cytotoxicity in BxPC-3 Cells

**Figure 1:**
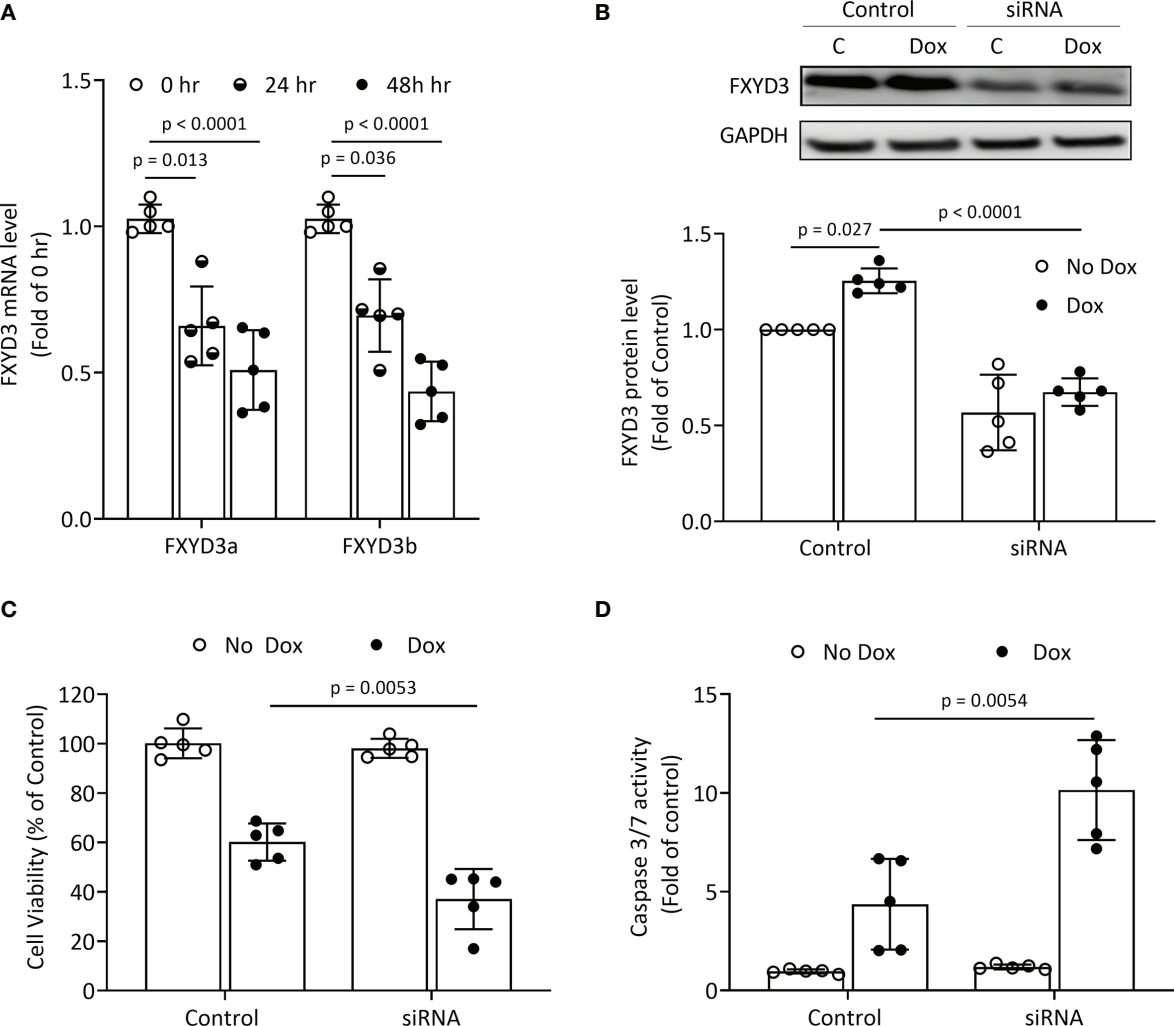
FXYD3-siRNA transfection of BxPC-3 cells. **(A)** Expression of FXYD3a and FXYD3b mRNA after exposure of cells to FXYD3 siRNA for 24 or 48 h **(B)** Effect of siRNA and Dox on FXYD3 expression. Cells were exposed to 1 μM Dox or Dox-free culture medium for 48 h after transfection as indicated. **(C)** FXYD3 siRNA transfection and effect of Dox on cell viability. Cells were exposed to Dox or Dox-free culture medium as for **(C)**. **(D)** FXYD3 siRNA transfection and effect of Dox on Caspase-3/7 activity.

BxPC-3 pancreatic cancer cells which have high FXYD3 expression were treated with siRNA transfection to modulate FXYD3 expression. **Figure 1A** shows that the siRNA reduced levels of endogenous FXYD3a and FXYD3b mRNA, two splice variants of FXYD3, at 24 and 48 h after treatment when compared with a control. Transfection with siRNA also decreased the protein expression of FXYD3 in BxPC-3 cells by ~60% after 48 h (**Figure 1B**).

FXYD3 siRNA transfection alone had no significant effects on viability or caspase 3/7 activation in BxPC-3 cells. When co-treated with Dox, transfection augmented a decreased viability (**Figure 1C**) and an increased caspase 3/7 activation (**Figure 1D**). The siRNA-directed suppression of FXYD3 protein sensitized BxPC-3 cells to Dox.

### Effects of Novel FXYD3 Derivatives on Protein Glutathionylation

**Figure 2:**
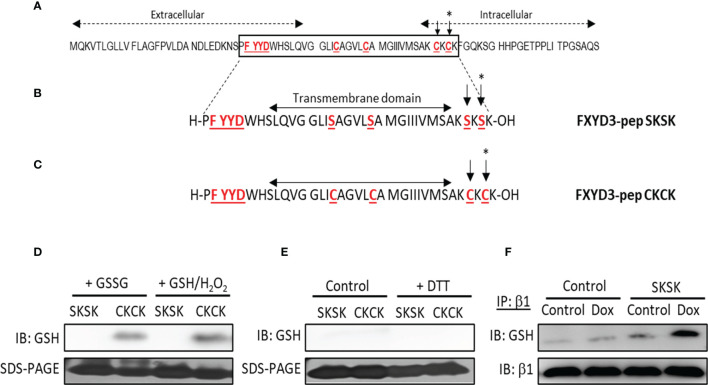
Amino acid sequences of FXYD3 and its derivatives. **(A)** Wild-type (WT) FXYD3. Cys residues mutated to Ser in full-length recombinant protein and the FXYD motif in the extracellular domain are shown in red font and are underlined. A Cys residue (indicated by *) in the cytoplasmic domain near the inner membrane leaflet and bracketed by basic amino acids (here Lys) is obligatory for susceptibility to glutathionylation of members of the FXYD protein family. Such susceptibility is critical for a FXYD protein, including FXYD3, to counter glutathionylation of the β1 Na^+^/K^+^-ATPase subunit. **(B)** Amino acid sequence of FXYD3-pep SKSK. Ser residues (indicated by *) that replaced Cys residues in the corresponding sites in WT FXYD3 are indicated in red font. **(C)** Amino acid sequence of FXYD3-pep CKCK that has Cys residues (indicated by *) of WT FXYD3 retained. **(D)** Immunoblots for GSH indicating glutathionylation of FXYD3-pep CKCK but not FXYD3-pep SKSK with exposure to GSSG or GSH/H_2_O_2_. **(E)** Reversibility of FXYD3-pep CKCK glutathionylation with exposure to DTT. **(F)** Immunoblot for GSH of cell lysate immunoprecipitated for the β1 Na^+^/K^+^-ATPase subunit after exposure to Dox. Pre-exposure to FXYD3-pep SKSK augments Dox-induced β1 Na^+^/K^+^-ATPase subunit glutathionylation.

The amino acid sequence of wild-type (WT) FXYD3 is shown in **Figure 2A**. The peptide analogue of the Cys-mutated FXYD3 protein with poorly conserved intracellular and extracellular domains eliminated FXYD3-pep SKSK, as shown in **Figure 2B**, and the analogue with Cys residues of the WT protein retained FXYD3-pep CKCK, as shown in **Figure 2C**. As expected, immunoblotting with a GSH antibody identified glutathionylation of FXYD3-pep CKCK but not of FXYD3-pep SKSK (**Figure 2D**, one of two similar experiments is shown). Dithiothreitol (DTT) is a reducing agent, which is expected to decrease existing glutathionylation. When incubated with DTT, the signal for glutathionylation of FXYD3-pep CKCK was eliminated (**Figure 2E**).

The effects of FXYD3-pep SKSK on glutathionylation of the β1 Na^+^/K^+^-ATPase subunit was examined in BxPC-3 cells submitted to the oxidative stress induced by Dox. BxPC-3 cells were exposed to 1 µM FXYD3-pep SKSK for 2 h. The cells were washed and then incubated for 24 h in a solution that included only 1 µM Dox but was free of FXYD3-pep SKSK. The levels of glutathionylation of the Na,K-ATPase β1 subunit was quantified in the precipitate obtained in immunoprecipitation experiments with an anti-GSH antibody. Exposure to FXYD3-pep SKSK increased the signal for β1 Na^+^/K^+^-ATPase subunit glutathionylation (**Figure 2F**).

### FXYD3-pep SKSK Displaces WT FXYD3 From the α1 Na^+^/K^+^-ATPase Subunit

**Figure 3:**
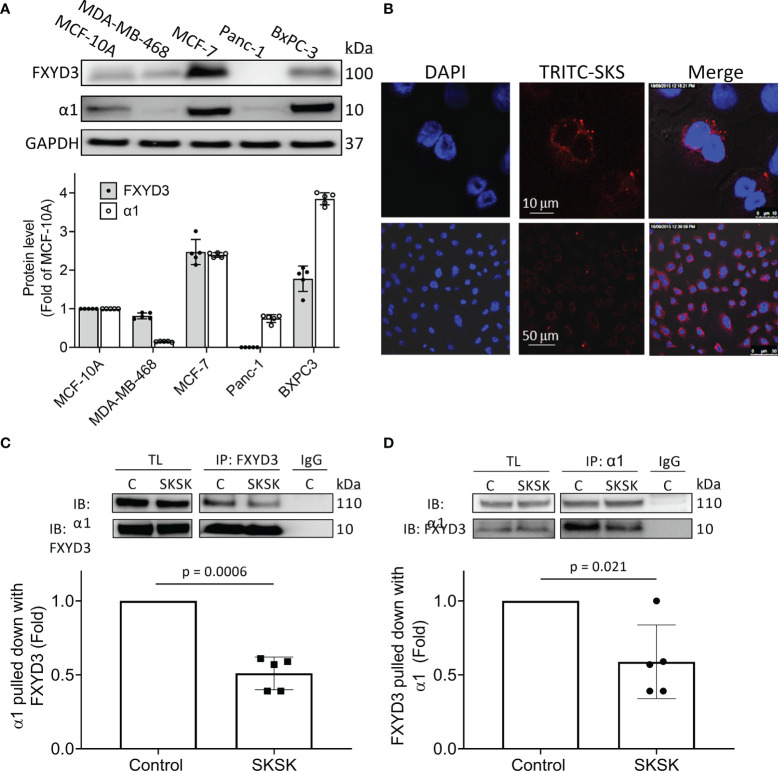
Distribution of FXYD3-pep SKSK and its ability to displace FXYD3 from the α1 Na^+^/K^+^-ATPase subunit. **(A)** FXYD3 and Na^+^/K^+^-ATPase α1 subunit expression in MCF-7 and MDA-MB-468 breast cancer cells and in BxPC-3 and Panc-1 pancreatic cancer cells. Expression is normalized to expression in human non-cancer MCF-10A cells; GAPDH was the internal loading control. **(B)** Immunofluorescence showing distribution of TRITC-labeled FXYD3-pep SKSK in BxPC-3 cells. Cells were exposed to 1 µM TRITC-tagged FXYD3-pep SKSK (TRITC-SKSK) (red) for 2 h and fluorescence microscopy performed 24 h after the peptide was washed off. DAPI was used to counterstain the nucleus (blue). One of 5 similar experiments is shown. Fluorescence is predominantly peri-nuclear. Scales are shown in the middle panel.** (C)** Immunoblot (IB) of α1 Na^+^/K^+^-ATPase subunit with WT FXYD3 immunoprecipitant in lysate of BXPC-3 with and without exposure of the cells to 1 μM FXYD3-pep SKSK for 2 h before lysis. **(D)** Immunoblot of WT FXYD3 with the α1 subunit immunoprecipitant in the lysate from the cells. C, control; TL, total lysate; non-immune IgG (IgG), negative control for IP. The efficiency of the Co-Ip can be estimated by the comparison of β1 subunit expression in the initial total lysate and the unbound supernatant after IP in BxPC-3 cells (data not shown). Approximate binding efficiency was ~90%.

Levels of FXYD3, normalized to levels in the non-transformed human breast cell line MCF-10A, were much higher in the BxPC-3 and MCF-7 cells than in the MDA-MB-468 and Panc-1 cells. Overexpression of the α1 Na^+^/K^+^-ATPase subunit broadly followed the same pattern as FXYD3 expression (**Figure 3A**). We did not detect WT FXYD3 protein or FXYD3 mRNA for Panc-1 (not shown), as others also reported (6).

We examined the cellular distribution of FXYD3-pep SKSK. For this, we used pancreatic BxPC-3 cells because they exhibited the strongest signal for expression of α1 Na^+^/K^+^-ATPase subunits (**Figure 3A**) that FXYD3-pep SKSK is expected to bind to. We exposed cells to TRITC-tagged FXYD3-pep SKSK for 2 h. The peptide was then washed off, and fluorescence microscopy was performed 24 h later.

TRITC-tagged FXYD3-pep SKSK was detected in the cytosol, particularly in the perinuclear region (**Figure 3B**). The fluorescent signal was not detected in cells exposed to FXYD3-pep SKSK without TRITC-tag (data not shown). A fluorescent signal that appears with a 2-h exposure to TRITC-tagged FXYD3-pep indicates that the peptide is membrane permeable, as expected for its amino acid sequence that mostly corresponds to the transmembrane domain of the FXYD3 protein (**Figure 2**). Persistence of the fluorescent signal 24 h after TRITC-tagged FXYD3-pep was washed off would not be expected unless the peptide binds intracellularly. A fluorescent signal reflecting binding to plasmalemmal Na^+^/K^+^-ATPase was not selectively detected. However, a signal from such fluorescence might be undetectable with overlapping of signals in multilayered cultured cells.

To examine if FXYD3-pep SKSK can displace WT FXYD3 from Na^+^/K^+^-ATPase, we exposed BxPC-3 cells to 1 µM FXYD3-pep SKSK for 2 h before cell lysis. We used an antibody directed against an epitope in WT FXYD3 that is absent in the shortened FXYD3-pep SKSK to detect the FXYD3 protein and found that FXYD3-pep SKSK reduced the co-immunoprecipitation (Co-Ip) of the Na^+^/K^+^-ATPase α1 subunit with FXYD3 by ~50% (**Figure 3C**). A similar reduction was observed in reverse Co-Ip (**Figure 3D**). The peptide displaced the native FXYD3 from the Na^+^/K^+^-ATPase α1 subunit.

### FXYD3-pep SKSK Enhances Dox-Mediated Cytotoxicity

We examined if displacement of WT FXYD3 from Na^+^/K^+^-ATPase is reflected in viability of cells exposed to FXYD3-pep SKSK with and without co-exposure to Dox for 48 h. The culture medium that contained FXYD3-pep SKSK and Dox was replaced with fresh medium every 24 h.

**Figure 4:**
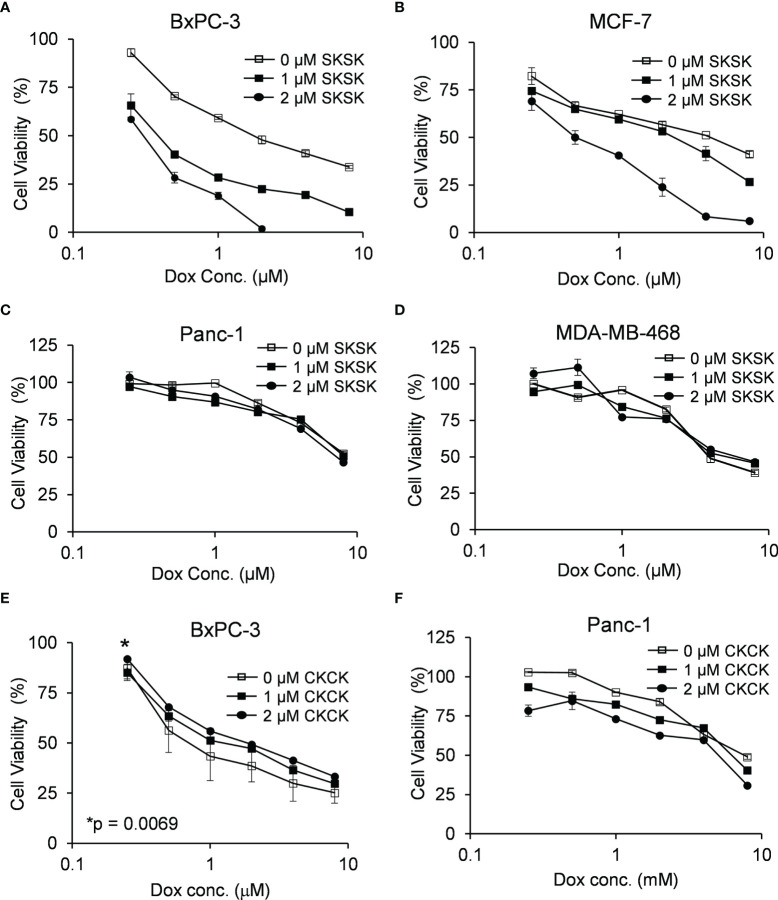
Effect of FXYD3-pep SKSK and FXYD3-pep CKCK on cell viability with co-exposure to Dox. Viability of **(A)** BxPC-3 cells, **(B)** MCF-7 cells. **(C)** Panc-1 cells, and **(D)** MDA-MB-468 cells when treated with Dox and 0, 1, or 2 µM FXYD3-pep SKSK for 48 h Viability of **(E)** BxPC-3 cells and **(F)** Panc-1 cells treated with Dox and FXYD3-pep CKCK for 48 h * in panel € refers to a significantly increased viability with exposure of cells to 2 µM FXYD3-pep CKCK compared with exposure to solutions free of the peptide. Results are from 5 experiments for each peptide concentration.

FXYD3-pep SKSK alone at 1 or 2 µM did not decrease cancer cell viability (data not shown). However, it augmented a Dox-induced decrease in viability of pancreatic BxPC-3 and breast MCF-7 cells that express high levels of FXYD3. For BxPC-3 cells, the IC50 for Dox was shifted from ~1.74 to ~0.4 μM and ~0.3 μM with exposure to 1 and 2 μM FXYD3-pep SKSK, respectively (**Figure 4A**). For MCF-7 cells, FXYD3-pep SKSK augmented the effects of Dox with a shift in IC50 from ~4.3 to ~0.5 μM with exposure to 2 µM FXYD3-pep SKSK (**Figure 4B**). FXYD3-pep SKSK did not augment the effect of Dox on pancreatic Panc-1 cells that do not express FXYD3 (**Figure 4C**) or on the breast cancer cell line MDA-MB-468 that expresses FXYD3 at a low level (**Figure 4D**). The peptide derivative that retained the Cys residues of the WT FXYD3 protein, FXYD3-pep CKCK, and reduced Dox induced cytotoxicity in BxPC-3 cells (**Figure 4E**) but had no effect on Panc-1 cells that do not express the FXYD3 protein (**Figure 4F**).

**Figure 5:**
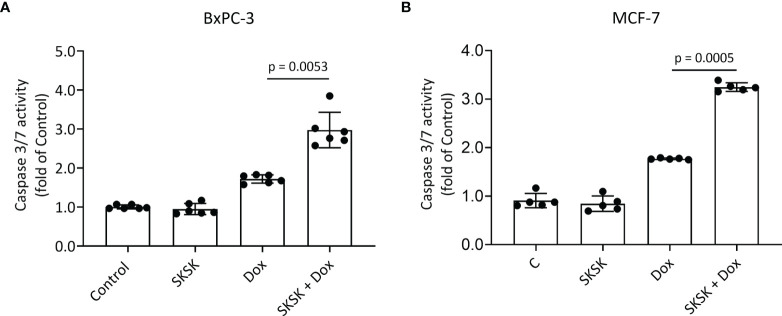
Effect of FXYD3-pep SKSK on caspase 3/7 activity. **(A)** BxPC-3 and **(B)** MCF-7 cells were exposed to solutions that included 1 μM FXYD3-pep SKSK or were peptide-free for 24 h and then for a further 48 h to solutions that included the peptide or were peptide-free and included Dox, 0.5 μM for the BxPC-3 cells, and 2.5 μM for the MCF-7 cells as indicated. Caspase 3/7 activity was used as index of apoptosis. N = 5.

Exposure of BxPC-3 cells (**Figure 5A**) and MCF-7 cells (**Figure 5B**) to FXYD3-pep SKSK alone had no effect on caspase 3/7 activity, but exposure to the peptide augmented an effect of Dox on caspase 3/7 in both cell lines.

### Plasmalemmal Na^+^/K^+^-ATPase Is an Unlikely Target for FXYD3-pep SKSK

**Figure 6:**
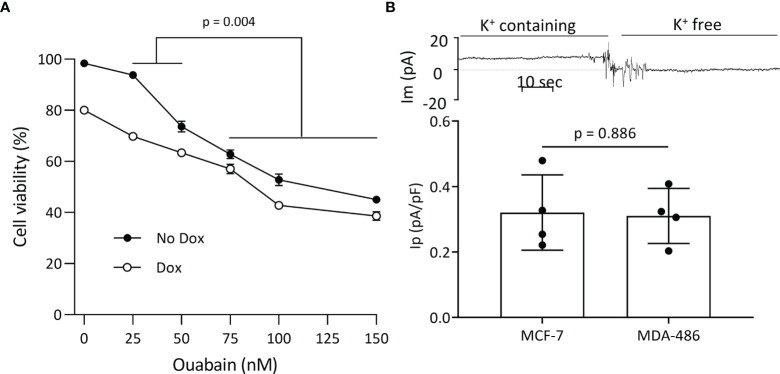
Plasmalemmal Na^+^/K^+^-ATPase as a potential target for FXYD3-pep SKSK. **(A)** MCF-7 cell survival after a 48-h exposure to ouabain in concentrations indicated, with or without co-exposure to 1 μM doxorubicin (Dox). * indicates difference in additive effect of ouabain and Dox at 25 or 50 nM ouabain vs. 70–150 nM ouabain. N = 5 for each ouabain concentration. **(B)** Electrogenic Na^+^/K^+^-ATPase pump currents (I_p_) of MCF-7 cells that do, and MDA468 cells that do not, overexpress Na^+^/K^+^-ATPase. The trace of membrane current (Im) was recorded in an MCF-7 cell before and after Na^+^-K^+^ pump activity was eliminated with exposure to K^+^-free extracellular solution. The inward shift to a near-zero holding current in K^+^-free solution identifies I_p_. Currents were sampled with an electronic cursor after electrical noise caused by extracellular solution change had subsided.

Plasmalemmal Na^+^/K^+^-ATPase is critical for cell survival, and since WT FXYD3 protects the Na^+^/K^+^-ATPase against inhibition induced by oxidative stress (2), we examined if direct inhibition of the Na^+^/K^+^-ATPase reproduces the effect of FXYD3-pep SKSK in augmenting Dox-induced cytotoxicity. We exposed MCF-7 breast cancer cells to ouabain that, due to its hydrophilicity, is poorly membrane-permeable and inhibits plasmalemmal Na^+^/K^+^-ATPase at extracellular sites (21). Cells were exposed to ouabain with or without co-exposure to Dox. Ouabain alone reduced cell viability in a concentration-dependent manner. However, a modest augmentation of Dox-induced cytotoxicity decreased with an increase in ouabain concentration (**Figure 6A**), in contrast to the increased augmentation of Dox cytotoxicity with an increase in the concentration of FXYD3-pep SKSK from 1 to 2 µM (**Figure 4**).

The different levels of Na^+^/K^+^-ATPase subunit expression might reflect a difference in the cells’ functional plasmalemmal Na^+^/K^+^-ATPase. FXYD3 and α1 Na^+^/K^+^-ATPase subunits are markedly upregulated in MCF-7 cells that are sensitized to Dox cytotoxicity by FXYD3-pep SKSK relative to levels in the MDA-MB-468 cells that are not sensitized (**Figure 3A**). We measured I_p_ of the two cell lines. There was no difference in I_p_ between the cells (**Figure 6B**). The differences in α1 Na^+^/K^+^-ATPase subunit expression between MCF-7 and MDA-MB-468 cells but similar plasmalemmal Na^+^/K^+^-ATPase pump capacity implicate an intracellular location of overexpressed Na^+^/K^+^-ATPase in the cells that are sensitized to Dox with exposure to FXYD3-pep SKSK.

## Discussion

Similar to the distribution of overexpressed WT FXYD3 in resected pancreatic (6) and breast cancers (8), we detected TRITC-tagged FXYD3-pep SKSK intracellularly, predominately in a perinuclear distribution. Immunofluorescence signals for WT FXYD3 in MCF-7 cells partly overlap with signals for Na^+^/K^+^-ATPase α subunits (8), and a mostly perinuclear distribution of the β1 Na^+^/K^+^-ATPase subunits in cancer cells is also reported with the development of some colorectal cancers (22). We are not aware of studies reporting overexpression of the intact α/β/FXYD3 Na^+^/K^+^-ATPase molecular complexes in the intracellular compartment in cancers. However, all its constituents are reported intracellularly and in a shared distribution.

FXYD3 in MCF-7 cells is expressed in the plasmalemma as well as in intracellular membrane compartments (8). The overexpressed FXYD3 is confined to the perinuclear region because the stem cell-related transcription factor SOX9 promotes FXYD3 transcription, and the synthesized FXYD3 protein in turn confines SOX9 to the nucleus in a regulatory feedback loop for FXYD3 amplification (23). With experimentally induced synthesis of FXYD3 in Chinese Hamster Ovary (CHO) cells, detectable FXYD3 becomes distributed in the nuclear envelope and the endoplasmic reticulum (ER) but not in the plasmalemma (24). Here, it is unlikely that FXYD3-pep SKSK sensitized the cells to Dox because of a dependence on a high catalytic plasmalemmal Na^+^/K^+^-ATPase activity. The high expression of WT FXYD3 in parallel with the expression of α1 subunits in MCF-7 cells relative to MDA-MB-468 cells was not reflected by different maximally activated I_p_. Furthermore, while MDA-MB-468 cells did not overexpress WT FXYD3, they did express the protein at levels comparable to those for non-transformed human breast cells, yet FXYD3-pep SKSK did not sensitize MDA-MB-468 cells to Dox.

Facilitated by bonds from FXYD proteins to α and β subunits seen in the crystal structure (4), the primary overexpression of FXYD3 in cancer cells might lead to an intracellular accumulation of FXYD3/α/β Na^+^/K^+^-ATPase complexes. That such assembly plausibly occurs is indicated by the spontaneous formation of FXYD/α/β Na^+^/K^+^-ATPase complexes with the *in vitro* exposure of human α1/β1 subunits to a molar excess of FXYD1, FXYD2, or FXYD3. FXYD/α/β Na^+^/K^+^-ATPase complexes also assemble when rat FXYD1, FXYD2, or FXYD4 is co-expressed with rat α1 subunits in HeLa cells, indicating that the effects shown for purified human proteins are biologically relevant in intact cells (25).

That an excess of FXYD proteins stabilizes the Na^+^/K^+^-ATPase structure and function is supported by how recombinant full-length WT FXYD3 prevents a decrease in α1/β1 Co-Ip detected in cardiac myocyte lysate and prevents a decrease of I_p_ measured in intact voltage-clamped myocytes during oxidative stress (2). However, while WT- and Cys-mutated recombinant proteins in equimolar concentrations similarly displace the FXYD1 native to myocytes, only the WT FXYD3 has these preventative effects. The proteins share amino acids that have bonds to α- and β subunits, and presence of Cys residues rather than displacement of WT proteins per se accounts for their effects on integrity and function of Na^+^/K^+^-ATPase.

Even with the overexpression of WT FXYD3 in BxPC-3 cells, FXYD3-pep CKCK had a protective effect against Dox-induced cytotoxicity while FXYD3-pep SKSK augmented it. The two peptides share with their full-length equivalent proteins all domains with bonds to the α/β Na^+^/K^+^-ATPase complex. However, Cys residues, including the specific residue that mutational studies indicate confers susceptibility of FXYD proteins to glutathionylation, had been replaced by Ser in FXYD3-pep SKSK (**Figure 2**) and it is a FXYD protein’s susceptibility to glutathionylation that counters glutathionylation of the β1 subunit (2). Effects of the peptides here indicate that augmentation of cytotoxicity with siRNA-induced downregulation of FXYD3 (9) reflects the role of the Cys residue that is critical for countering β1 subunit glutathionylation.

With selective inhibition of plasmalemmal Na^+^/K^+^-ATPase at ouabain concentrations >50 nM, there was no dose-dependent increase in ouabain’s augmentation of Dox’s cytotoxicity; that is, ouabain did not reproduce the large dose-dependent augmentation of Dox’s cytotoxicity that FXYD3-pep SKSK induced. Therefore, while glutathionylation of the β1 subunit inhibits Na^+^/K^+^-ATPase (26), selective inhibition of plasmalemmal Na^+^/K^+^-ATPase ion transport is unlikely to account for the pattern of sensitization to Dox seen with FXYD3-pep SKSK.

In addition to mediating ion transport, Na^+^/K^+^-ATPase facilitates cell adhesion *via* intercellular β1–β1 subunit bridges, and exposure to ouabain in nanomolar concentrations augments such cell adhesion in CHO fibroblasts overexpressing β1 subunits (27). Ouabain also prevents β1 subunit glutathionylation induced by oxidative stress, shown for intact cardiac rabbit myocytes and isolated pig kidney Na^+^/K^+^-ATPase-enriched membrane fragments (28). β1 subunit glutathionylation and α1/β1 subunit Co-Ip are inversely related (28), and molecular dynamics simulations suggest that this might reflect the structural disruption that a GSH adduct causes between transmembrane domains of the glutathionylated heterodimer, combined with a disruption of the cytosolic membrane leaflet necessary for access of the cytosolic GSH (29).

FXYD3 from intracellular α/β/FXYD3 Na^+^/K^+^–ATPase complexes in cells overexpressing FXYD3 might be a source that counters glutathionylation of β1 subunits in plasmalemmal Na^+^/K^+^-ATPases. The effect such a source of FXYD proteins can have is indicated by the reversal of decreased myocyte I_p_ induced by oxidative stress when recombinant FXYD1 or FXYD3 proteins are included in patch pipette solutions perfusing the intracellular compartment. A decrease in the β1 subunit’s glutathionylation in parallel with an increase in its Co-Ip with α1 subunits in lysates of myocytes supported FXYD protein-dependent protection of α1/β1 heterodimer integrity accounting for effects on ion transport (2).

Since intercellular β1–β1 subunit bridges extend to the cytoskeleton through the binding of β1 to α1 subunits, that in turn have bonds to the cytoskeleton (30), disruption of the α1/β1 heterodimer with glutathionylation is expected to disrupt cell adhesion. Conversely, overexpressed FXYD3 might counter such disruption. Consistent with this, FXYD3 knockdown impairs growth and disperses cells in MCF-7 tumors expressed in mice (23), and in human colon adenocarcinoma Caco-2 cells, FXYD3 knockdown decreases the transepithelial electrical resistance of confluent cell layers consistent with decreased intercellular adhesion (31).

In contrast to FXYD3 promoting cell adhesion, transfection of a mouse kidney cell line with FXYD5 reduces adhesion, as indicated by a decrease in transepithelial electrical resistance (32). However, this is expected if disruption of the α1/β1 heterodimer with glutathionylation disrupts cell adhesion because FXYD5 has a Gln residue (33) at the site where a Cys residue bracketed by basic amino acids is obligatory for a FXYD protein to counter glutathionylation of the β1 subunit. A single, selective mutation of the residue (Cys→Ala) eliminates an FXYD protein’s effect to counter β1 subunit glutathionylation (2).

Disruption of cell adhesion with FXYD5 transfection was attributed to a decrease in glycosylation of the β1 subunit’s extracellular domain (32), and inhibiting glycosylation can augment the cytotoxicity of Dox (34). FXYD5 is functionally a WT protein equivalent of FXYD3-pep SKSK in terms of their expected effects on glutathionylation of β1 subunits, and decreased glycosylation or increased glutathionylation of the subunits is not mutually exclusive in potential effects on subunit-dependent intercellular adhesion. The complexity of β1 subunits Na^+^/K^+^-ATPase *N-*glycans is also important for effects of the subunit on intercellular adhesion (35). However, an effect of FXYD3-pep SKSK on this cannot be invoked at present.

The FXYD peptide derivatives here augment key functions of the WT proteins they are derived from or, with a Cys-mutated modification, eliminate the function by displacing the WT protein from its only known binding partner, Na^+^/K^+^-ATPase. Exposure over minutes–hours avoids confounding cellular adjustments with transfection to overexpress FXYD proteins or silencing to reduce their abundance over days–weeks, and, in contrast to the experimental use of recombinant FXYD proteins (2), purified peptides are easy and inexpensive to make.

The FXYD3 peptide derivatives we report here are useful tools for exploring the role of overexpressed FXYD3 in cancer and its potential as a treatment target. With further modifications, FXYD3-pep SKSK might be developed into a treatment sensitizer. However, one caveat must be kept in mind. The peptide is, at least in part, expected to be a functional equivalent of FXYD5, and FXYD5 expression increases the metastatic potential in a mouse breast cancer model. This is associated with a decrease in plasmalemmal Na^+^/K^+^-ATPase β1 subunit expression and a decrease in intercellular adhesion (36). FXYD5 expression also appears to increase the metastatic potential of human cancers (37).

While the focus here is on FXYD3-pep SKSK, the protective effect of FXYD3-pep CKCK against Dox-induced cytotoxicity reflects properties that also may be useful. The peptide retains the Cys residue and, in turn, an ability to protect integrity and function of the α1/β1 Na^+^/K^+^-ATPase heterodimer (2). A potential use is exemplified by treatment of acute respiratory distress syndromes, including SARS-CoV-2 infection. Reduced Na^+^ export from alveolar cells mediated by Na^+^/K^+^-ATPase and compromised β1 subunit-dependent integrity of the alveolar epithelial barrier are implicated in the pathophysiology of pulmonary alveolar edema (38, 39). A peptide drug based on FXYD3-pep CKCK and modified to resist *in vivo* breakdown might offer an approach to treatment.

## Data Availability Statement

The original contributions presented in the study are included in the article/**Supplementary Material**. Further inquiries can be directed to the corresponding authors.

## Author Contributions

C-CL contributed to the design and implementation of the research, to the analysis of the results, and to the writing of the manuscript. YK, RT, AG, AW, and EH performed the experiments. FC, RB, and HR aided in interpreting the results and worked on the manuscript. All authors contributed to the article and approved the submitted version.

## Funding

The work is supported by a grant from the Avner Pancreatic Cancer Foundation, Heart Research Australia, and Ramsay Research and Teaching Fund (Australia). YK was supported by Research Training Program Stipend Scholarship (Australia) and a Ramsay research top up scholarship (Australia). AG was supported by a UTS Chancellors Postdoctoral Research Fellowship (Australia). EH was supported by Heart Research Australia.

## Conflict of Interest

The authors declare that the research was conducted in the absence of any commercial or financial relationships that could be construed as a potential conflict of interest.

## Publisher’s Note

All claims expressed in this article are solely those of the authors and do not necessarily represent those of their affiliated organizations, or those of the publisher, the editors and the reviewers. Any product that may be evaluated in this article, or claim that may be made by its manufacturer, is not guaranteed or endorsed by the publisher.
