# Supplementary material for: Displacement of Native FXYD Protein From Na+/K+-ATPase With Novel FXYD Peptide Derivatives: Effects on Doxorubicin Cytotoxicity
Source: Front Oncol. 2022 Mar 17;12:859216. doi: 10.3389/fonc.2022.859216 (PMC8968713; doi:10.3389/fonc.2022.859216)
Supplement: Supplementary file 1 [file Image_1.pdf]

Supplemental Figure. S1

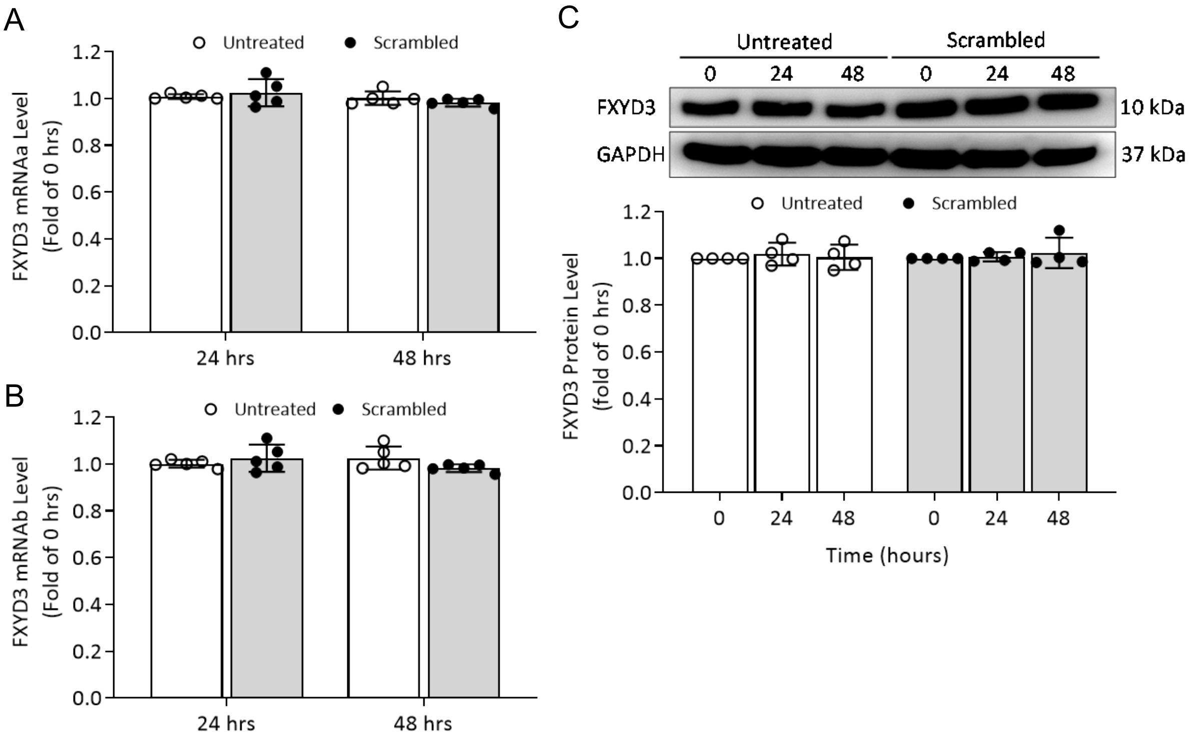

Figure. S1. FXYD3 mRNA and protein expression level. (A) FXYD3a and **(B)** FXYD3b mRNA and **(C)** FXYD3 protein level in untransfected (Untreated) and non-silencing (Scramble) siRNA treated BXPC-3 cells after 24 hr and 48 hr FXYD3 siRNA transfection. All data are shown as mean ±SD of 4-5 experiments.
